# Lysine Dendrigraft Nanocontainers. Influence of Topology on Their Size and Internal Structure

**DOI:** 10.3390/pharmaceutics10030129

**Published:** 2018-08-13

**Authors:** Boris Okrugin, Maxim Ilyash, Denis Markelov, Igor Neelov

**Affiliations:** 1 Faculty of Physics, St. Petersburg State University, Ulyanovskaya Str.1, Petrodvorets, 198504 St. Petersburg, Russia; b.okrugin@spbu.ru (B.O.); d.markelov@spbu.ru (D.M.); 2 St. Petersburg National University of Informational Technologies, Mechanics and Optics (ITMO University), Kronverksky pr.49, 197101 St. Petersburg, Russia; ilyashmu@gmail.com

**Keywords:** poly-l-lysine, dendrigrafts, molecular dynamics simulation

## Abstract

Poly-l-ysine dendrigrafts are promising systems for biomedical applications due to their biodegradability, biocompatibility, and similarity to dendrimers. There are many papers about the use of dendrigrafts as nanocontainers for drug delivery. At the same time, the number of studies about their physical properties is limited, and computer simulations of dendrigrafts are almost absent. This paper presents the results of a systematic molecular dynamics simulation study of third-generation lysine dendrigrafts with different topologies. The size and internal structures of the dendrigrafts were calculated. We discovered that the size of dendrigrafts of the same molecular weight depends on their topology. The shape of all studied dendrigrafts is close to spherical. Density profile of dendrigrafts depends on their topology.

## 1. Introduction

**Figure 1:**
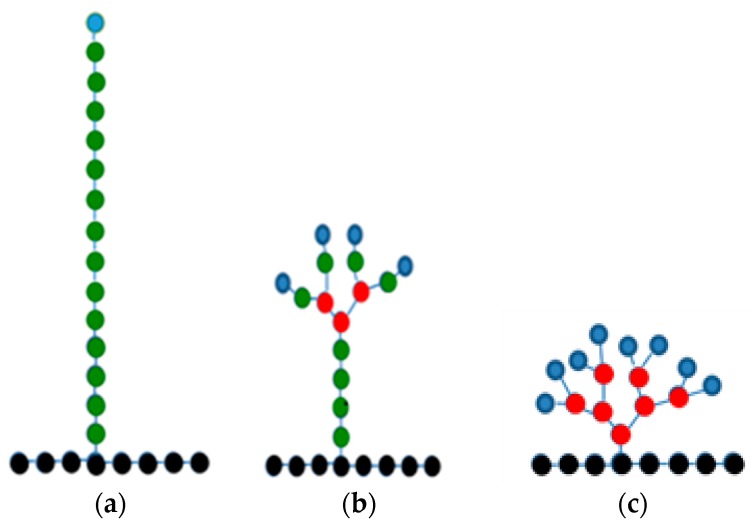
Three different lysine dendrigrafts: (**a**) dendrigraft 1; (**b**) dendrigraft 2; and (**c**) dendrigraft 3 with eight lysine residues in each main chain (core) marked by black points and 15 lysine residues (marked by green, red and blue points) in each of the eight side chains. Only one of the eight side chains is shown in Figure 1a–c for clarity: (**a**) dendrigraft 1, with 14 intermediate lysine residues (green), without branching lysine (red), and with one terminal lysine (blue) in each side chain; (**b**) dendrigraft 2, with eight intermediate lysines (green), three branching points (red), and four terminal lysine residues (blue) in each side chain; (**c**) dendrigraft 3, without intermediate lysine residues (green) and with seven branching points (red) and eight terminal lysine residues (blue) in each side chain.

Nowadays, there are many examples of the use of highly branched molecules—such as dendrimers, dendronized polymer brushes, dendrigrafts, and hyperbranched polymers—for various industrial and biomedical applications. Dendrigrafts are very similar to regular dendrimers, but they have a linear chain-like core instead of the usual point-like core in dendrimers. They also are slightly less regular than dendrimers. The main advantage of the dendrigrafts is the low cost of their synthesis compared with the cost of dendrimers. One of the key features of the dendrimers is that the number of the terminal functional groups on the periphery increases exponentially with the generation number [1]. For this reason, dendrimers have many terminal groups available for functionalization, making them very popular for use in various nano-applications [2,3,4,5,6]. Biocompatible and biodegradable dendrimers and dendrigrafts could be widely used in biomedical applications. Peptide dendrimers [7,8,9,10,11,12,13,14] and dendrigrafts [15,16,17,18] consisting of amino acid residues are important examples of such dendrimers and dendrigrafts. In particular, lysine dendrigrafts of all generations have eight lysine residues in their core (see black circles in Figure 1). The poly-l-lysine PLL dendrigrafts are biodegradable [15], have low cellular toxicity [16], and are nonimmunogenic [17]. For further information about the applications of dendrigrafts, see references in paper [18].

Despite the popularity of PLL dendrimers and dendrigrafts in applications, there are only a small number of experimental and theoretical papers devoted to the systematic study of physical properties and computer simulations of PLL dendrimers [19,20,21,22,23] and almost no papers on theory and computer simulations of PLL dendrigrafts [18,24,25]. Our aim is to study the influence of the topology of the side chains on the size and internal structure of three third-generation dendrigrafts by using a molecular dynamics simulation method.

## 2. Materials and Methods

We used a symmetrized model of third-generation lysine dendrigrafts in which all eight monomers of dendrigraft are the same. We constructed three monomers of different topologies. Each of the eight monomers of dendrigrafts contains one lysine residue in the main chain (marked in black in Figure 1a–c) and 15 lysine residues in the side chain (i.e., each of the eight dendrigraft monomers consists of 16 lysine residues). Each lysine residue in the side chain is either a branching point (marked in red in Figure 1), internal segment (green), or terminal segment (blue).

All three types of monomers have the same molecular weight M (see Table 1), but they have different topologies (number of branching points (marked by red in Figure 1a–c)) in side chains and number of terminal groups Nt (marked by blue) (see Figure 1 and Table 1). The side chain of the first monomer (of dendrigraft 1) is linear and thus does not have branching points. The side chain of the second monomer (of dendrigraft 2) has three branching points, and the side chain of the third monomer (of dendrigraft 3) has seven branching points. The branching points in Figure 1 (red) have no charge, each intermediate lysine residue (green) has one charge, and each terminal residue (blue) has two charges. After construction of the three different monomers and minimization of their energies, we connected them into a linear homopolymer chain consisting of eight monomers (using peptide bonds between circles marked by black in Figure 1). There are 16 lysine residues in each monomer of the third-generation dendrigraft (both in the main and side chain) and thus there are 128 lysine residues in the dendrigrafts as a whole. The total charge of each dendrigraft monomer will be equal to +16 and thus the total charge of all studied dendrigrafts consisting of 8 monomers will be equal to +16 × 8 = +128.

The Gromacs-4.5.6 package [26] with AMBER99SB-ildn force field [27] was used in the simulations of all systems. Electrostatic interactions were calculated using the particle mesh Ewald (PME) method. The simulations of each dendrigraft consisted of the construction of an initial conformation of a system, minimization of its energy, equilibration of the system, and molecular dynamic (MD) simulation during 720 ns. Dendrigrafts were studied in a water solvent (TIP3P model) and Cl^−^ counterions were added for compensation of dendrigraft charge. All calculations were performed at a temperature of 300 K and a pressure of 1 atm.

Three systems consisting of dendrigrafts with the same molecular weight, but with different side chain topology (and, as a result, with different side chain contour lengths and charge distributions), were constructed. To avoid interatomic overlap, additional energy minimization was performed for whole systems. The initial 600 ns were used in MD simulations for equilibration of the systems and the last 120 ns were used as productive runs for calculating the average values and distribution functions of different values (size, end-to-end distance, radial density, and charge distributions).

## 3. Results

### 3.1. Large-Scale Properties

**Figure 2:**
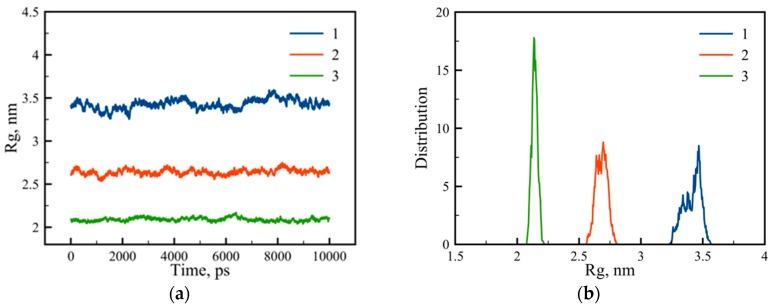
(**a**) The variation of the gyration radius *R*_g_ with time t in picoseconds and (**b**) the distribution function for dendrigrafts 1, 2, and 3.

The size of dendrigrafts can be calculated by instant mean-square gyration radius

(1) $$R_{g}{}^{2}=\frac{1}{M}\underset{i}{\sum }\left(m_{i}r_{i}{}^{2}\right),$$

where *r_i_* is the distance between the *i*-th atom and center of mass (COM) of the dendrigraft, and *m_i_* and M are the molecular mass of the *i*-th atom and all atoms of dendrigraft correspondingly. Summations were taken over all atoms of the dendrigraft. Time dependences of this value for all dendrigrafts are presented in Figure 2a. The averaging along the trajectory (during the last 120 ns of simulation) gives the mean square value of the *R*_g_. For dendrigraft 1 *R*_g_ = 3.42 nm, for dendrigraft 2 *R*_g_ = 2.64 nm, and for dendrigraft 3 *R*_g_ = 2.09 nm. Thus, the increase of the branching degree from dendrigraft 1 to dendrigraft 3 leads to smaller dendrigraft size due to the decrease of the contour length of the side chains (see Figure 1). We can compare values of *R*_g_ obtained from our simulation with *R*_g_ calculated from experimental hydrodynamics radius *R*_h_ [28,29] using theoretical connection of *R*_g_ and *R*_h_ for undrained spherical molecules:

(2) $$\frac{R_{g}}{R_{h}}=\sqrt{\frac{3}{5}}$$

The values of *R*_g_ calculated from MD simulations of dendrigraft 2 and dendrigraft 3 (2.64 nm and 2.09 nm, respectively) belong to the interval of values *R*_g_ = 1.65–2.65 nm (*R*_h_ = 2.14–3.43 nm) obtained by different experimental methods in [28,29], while our value of *R*_g_ for dendrigraft 1 is essentially higher than for the experimental ones.

The distribution function of the gyration radius is presented in the Figure 2b. One can see that distribution becomes increasingly narrower for dendrigrafts with higher numbers of branching points.

**Figure 3:**
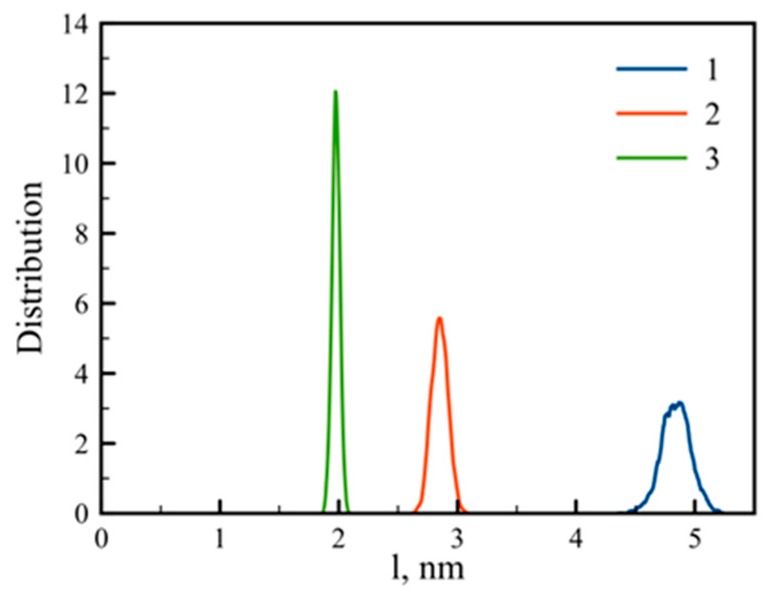
Distribution of side chain end-to-end distance (distance between the C_α_ atom of the main chain and terminal NH_3_^+^ group of the corresponding side chain) for dendrigrafts 1, 2, and 3.

Another parameter that characterizes the dendrigraft is the end-to-end distance of the side chain, or the distance between the C_α_ atoms of the lysine residue in the main chain and the corresponding terminal NH_3_^+^ group (blue circles in Figure 1) of each of the eight side chains. The distribution function of these values is presented in Figure 3. The result is quite predictable because increasing the number of branching points from dendrigraft 1 to dendrigraft 3 leads to a decrease in the contour length of the side chains.

**Figure 4:**
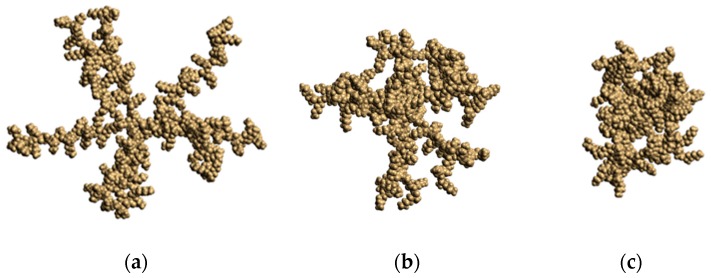
Snapshots of dendrigrafts: dendrigraft 1 (**a**); dendrigraft 2 (**b**); and dendrigraft 3 (**c**).

Figure 4 shows snapshots of dendrigrafts simulated in this paper. It is easy to see that, similar to dendrimers [30,31,32], all studied dendrigrafts have a close to spherical shape despite their linear core. Due to this reason, we could calculate radial distribution of internal characteristics of these molecules, including the radial density and charge distributions around the center of mass of the dendrigrafts.

### 3.2. Internal Structure

**Figure 5:**
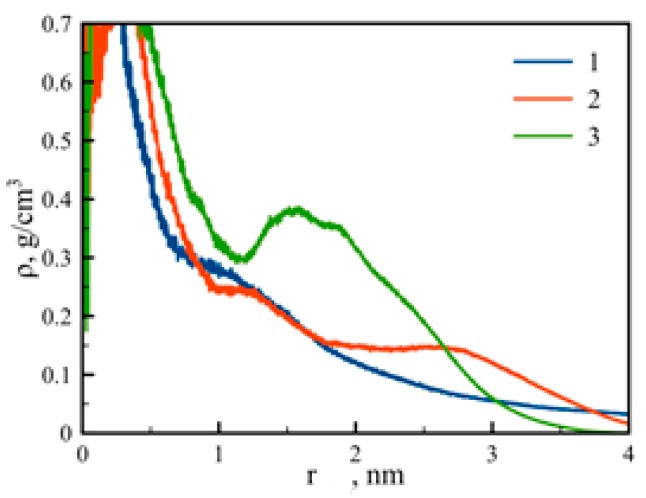
Normalized density profiles for dendrigrafts 1, 2, and 3 of generations G = 3, where *r* is the radial distance from the dendrigraft’s center of mass.

The dendrigraft internal structure can be characterized by the density profiles of atoms *ρ*(*r*) as:

(3) $$\rho \left(r\right)=\frac{1}{4\pi r^{2}}\overset{N_{a}}{\underset{i}{\sum }}m_{i}\delta \left(r-r_{\mathrm{com}}\right)$$

where *N*_a_ is the number of atoms, *m_i_* is the mass of *i*-th atom, and *r*_com_ is the position of the dendrigraft’s center of mass. The density profiles of the dendrigrafts are presented in Figure 5. It is easy to see that the density profiles of dendrigraft 1 and dendrigraft 2 are monotonous functions of distance *r* from the dendrigraft’s center of mass. Thus, the profiles for dendrigraft 1 and dendrigraft 2 correspond to the dense-core and loose-shell model of dendrimers. It was shown earlier that this model was valid, for example, for such dendrimers as PAMAM and polysiloxane dendrimers [33,34,35,36,37].

**Figure 6:**
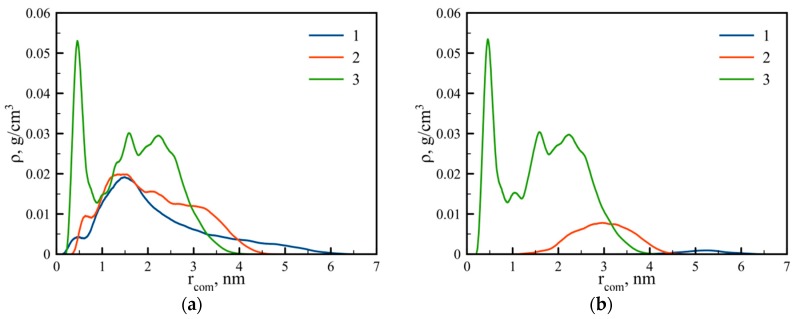
Distribution of (**a**) all charged NH_3_^+^ groups and (**b**) terminal charged NH_3_^+^ groups for dendrigrafts 1, 2, 3.

However, in the case of dendrigraft 3, there is a minimum of density profile (cavity) at a distance near 1 nm from its center of mass similar to that obtained for non-regular dendrigrafts in [18]. It was shown earlier that the presence of the cavity could be due to the segregation effect between the dendrimer segments [38,39,40]. In the case of dendrigraft 3, this segregation can occur between internal non-charged and terminal charged monomers. This hypothesis is confirmed by the shape of distribution function of charged groups in dendrigraft 3 (Figure 6). It is easy to see that in the region of minimum density, there is also a minimum in charge density. It is obvious that uncharged monomers are less hydrophilic than charged ones. Therefore, we assume that the cavity (a low-density hydrophobic region) in dendrigraft 3 could be used for the encapsulation of hydrophobic drugs and other hydrophobic molecules for their delivery to target cells or organs.

For dendrigrafts 1 and 2, the charged groups are distributed along the contours of the side chains. Because of this reason (and the larger contour length of the side chains), their radial distribution is wide enough (see lines 1 and 2 in Figure 6a). Figure 6b demonstrates the distribution of the terminal charged groups. Line 1 (blue), for dendrigraft 1, shows the distribution of only eight groups, which are the ends of each of the eight linear side chains. With the increasing branching degree from dendrimer 1 to dendrimer 2, the peak moves towards the center of the dendrimer and becomes higher due to a decrease of contour length and an increase of terminal groups in each side chain, from 8 to 32. Dendrigraft 3 in Figure 6b demonstrates even more pronounced behavior because the contour length decreases and the number of terminal groups increases from 32 to 128. There is also a noticeable back-folding effect because the terminal groups fold and penetrate toward the central region of the dendrigrafts.

**Figure 7:**
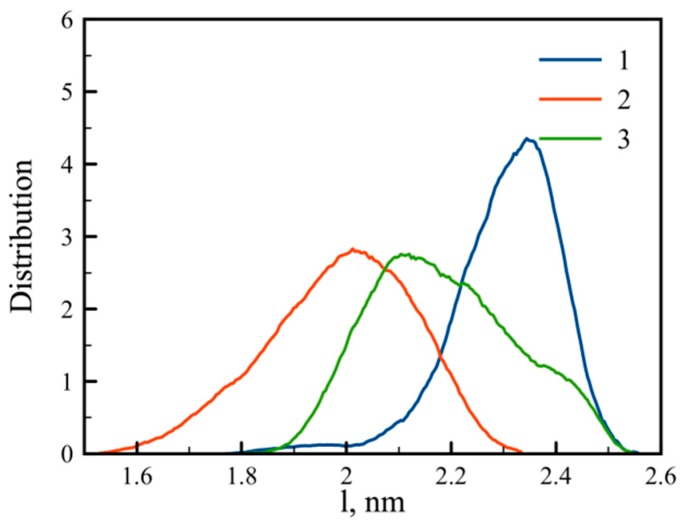
Distribution of end-to-end distance for dendrigrafts 1, 2, 3.

At the end we would like to check the distributions of end-to-end distances for the main chain (core region) of all dendrigrafts to understand if the main chain exists mainly in a coiled or in a stretched state. This distribution function is shown in Figure 7. It is easy to see that the distributions for all dendrigrafts are much wider than the corresponding distributions for end-to-end distances of side chains (see Figure 3). Thus the fluctuation of end-to-end distances is very large and the main chain is not always in the same state but passes through many possible states during simulation run.

In addition, note that the distribution for dendrigraft 3 (line 3) in Figure 7 is between the distributions for dendrigrafts 1 and 2. This means that the stretching of the main chain of the dendrigraft is not a monotonous function of the number of branching points in the side chain (or contour length of the side chain). The reason for this is not quite clear and we plan to discuss it in more detail in a future paper.

## 4. Discussion

Three third-generation PLL dendrigrafts with the same molecular weight and number of charged groups, but with different topology (0, 3 and 7 branching points in each side chain) were simulated in water by a full-atomic MD method. This method has been used in the past for simulation of lysine dendrimers [14,20,21,22,23,41] and lysine dendrigrafts [18,24,25]. In addition to Gromacs software, we used approaches and computer programs elaborated earlier in [42,43,44,45,46,47,48,49,50,51,52,53,54]. We have shown in the present paper that the size of dendrigraft depends on the branching point number. The higher the degree of branching, the smaller is the size of the dendrigraft. We found that sizes calculated from the simulations of dendrigrafts 2 and 3 (with three and seven branching points in each side chain, respectively) are in good agreement with the existing experimental data for PLL dendrigrafts [28,29]. At the same time, the size of dendrigraft 1 (with linear side chains) is essentially greater than that of experimental ones and it is likely that this topology is not realized in real lysine dendrigrafts.

We have shown that the radial density profile of a dendrigraft also depends on topology. In particular, for dendrigraft 1 and dendrigraft 2, the density profile is a monotonous function of the distance from the dendrigraft center of mass. For dendrigraft 3 (with a maximum number of branching points), there is a minimum of density profile at distances close to 1 nm from the dendrigraft’s center of mass. There is also a minimum of charge distribution at this distance. This means that, at these distances, there is a cavity (a low density hydrophobic area inside the dendrigraft). We think that this feature could be important for using this particular dendrigraft as a nanocontainer for hydrophobic drugs.

We observed a wide distribution of end-to-end distance of the main chain (linear core) for all dendrigrafts. This means that core fluctuations are large and a dendrigraft could change its shape significantly. We also found that the end-to-end distance of the main chain of dendrigraft core has non-monotonous dependence on the topology (branching point number). The reason for this behavior is not clear but will be studied in more detail in a future paper in which more types of dendrigrafts with different branching point numbers will be studied.

## Figures and Tables

**Table 1:** Parameters of the simulated dendrigrafts: M is the molecular weight and N_t_ is the number of terminal groups.

Type	M (g/mol)	N_t_
Dendrigraft 1	2947	8
Dendrigraft 2	2947	32
Dendrigraft 3	2947	64
